# From Willingness to Practice: A Person-Centered Study of Primary Teachers’ Critical-Thinking Readiness and the Enactment Gap

**DOI:** 10.3390/jintelligence14090215

**Published:** 2026-09-06

**Authors:** Zhen Qiang, Hong Yi, Zhuo Wang

**Affiliations:** 1 College of Education, Tianjin University, Tianjian 300350, China; zhen@tju.edu.cn; 2 College of Foreign Languages, Lanzhou Jiaotong University, Lanzhou 730070, China; 3 College of Foreign Languages, Hubei Minzu University, Enshi 445000, China; 4 College of Educational Sciences, Qingdao University, Qingdao 266070, China

**Keywords:** critical thinking, latent profile analysis, EFL teachers, teacher readiness, mixed methods, critical thinking disposition, individual differences

## Abstract

Critical thinking (CT) is a core competence that schools are expected to cultivate, yet whether it reaches classrooms depends on teachers’ readiness to teach it. Using an explanatory sequential mixed methods design, we surveyed 312 primary English-as-a-foreign-language teachers in Shandong Province, China, across seven dimensions of readiness and interviewed five teachers. Latent profile analysis identified four profiles organized by two weakly related dimensions—the capacity teachers bring to CT teaching and the constraints they perceive around them: Empowered-Engaged (15%), Willing-but-Constrained (28%), Moderate-Unobstructed (20%), and Committed-but-Under-resourced (37%). Endorsement of CT was near-universal, so the profiles differed in capacity and circumstance rather than in commitment. The central finding is a disconnection between what teachers believe and what they report doing: Willing-but-Constrained teachers wanted further professional learning as much as their Empowered-Engaged peers did, yet reported teaching CT less often. The divergence held under methods that account for classification uncertainty and under alternative measurement specifications, though not under all of them, and profile membership was largely unrelated to teachers’ background characteristics. The interview findings help explain why positive beliefs about CT did not always correspond to classroom practice. Teachers reported limited CT-specific training, examination pressure, time constraints, and norms that discouraged questioning, despite generally recognizing the value of CT. This suggests that the main barriers to CT instruction may lie not in teachers’ attitudes toward CT, but in their opportunities, preparation, and working conditions for implementing it.

## 1. Introduction

Critical thinking (CT)—the disposition and ability to analyze, evaluate, and reason toward well-grounded judgments—is increasingly understood not merely as an educational slogan but as a component of intelligence that predicts the quality of everyday, real-world decisions and can be both assessed and cultivated (Butler, 2024; Facione, 2000; Halpern & Dunn, 2021). It has become a defining aim of twenty-first-century schooling (Halpern, 1998). International curricular reforms increasingly position CT not as the province of a single subject but as a cross-curricular competence, and foreign-language classrooms are no exception: interpreting texts, weighing perspectives, and justifying claims are now widely regarded as integral to communicative competence rather than as distractions from it (Alnofaie, 2013; Ruano-Borbalan, 2023). Whether this aspiration reaches classrooms, however, depends far less on the wording of curricula than on the teachers who enact them, whose beliefs, competence, and working conditions ultimately determine whether CT is taught at all (Pajares, 1992; Reichert & Torney-Purta, 2019).

A large intervention literature demonstrates that CT is teachable. Dialogic discussion, problem-based and collaborative tasks, and explicit strategy instruction reliably improve students’ reasoning across age groups and disciplines (Abrami et al., 2015; Heijltjes et al., 2014; Xu et al., 2023; Y. Liu & Pásztor, 2022). Yet this body of work almost invariably presupposes teachers who are already willing and able to enact such pedagogy. The prior and more fundamental question—what makes a teacher ready to teach CT in the first place—has attracted comparatively little empirical attention, particularly in primary EFL settings, where teachers must reconcile higher-order thinking aims with the foundational demands of vocabulary, grammar, and communicative fluency, and typically do so under considerable examination pressure (Li, 2023; Zhang et al., 2020).

A further limitation is methodological. Most teacher-focused studies adopt a variable-centered approach, estimating average associations between antecedents such as attitudes or self-efficacy and outcomes across the full sample. In doing so, they implicitly treat teachers as a relatively homogeneous population whose differences are primarily quantitative. This assumption may be problematic if readiness instead reflects qualitatively distinct configurations of capacities and constraints. A variable-centered model, for example, cannot readily capture a teacher who is as motivated and knowledgeable as a highly effective colleague but works under substantially greater contextual constraints. When readiness is configural rather than simply dimensional, sample-level averages can conceal combinations of characteristics that are consequential for both classroom enactment and intervention. A person-centered approach is therefore needed to identify distinct readiness profiles and to determine which groups may require different forms of professional or institutional support.

The present study therefore adopts a person-centered approach. Latent profile analysis (LPA) classifies individuals into subgroups defined by shared configurations of characteristics, surfacing typologies that variable-centered methods do not directly reveal (Spurk et al., 2020). The readiness dimensions on which teachers are profiled follow the theory of planned behavior (Ajzen, 1991), in which attitude, subjective norm, and perceived behavioral control shape intention and behavior, with Bandura’s (1977) self-efficacy anchoring the control component.

Three gaps therefore motivate the present study. First, teacher readiness to teach CT has rarely been conceptualized as a configuration of co-occurring capacities and constraints rather than as a set of independent variables with average effects. Second, person-centered approaches, although well established in research on cognitive abilities and individual differences (Conte & Harmata, 2023) and increasingly used to study teacher wellbeing and engagement, have not yet been applied to CT readiness and remain uncommon in research on primary EFL teachers. Third, although discrepancies between teachers’ beliefs and practices are widely documented, existing research has not situated this belief–practice gap within a typology capable of identifying which teachers are most likely to experience it and under what conditions. Addressing these gaps requires a person-centered quantitative approach to identify distinct readiness profiles, complemented by qualitative inquiry to explain the mechanisms and contextual conditions underlying those profiles.

We then examine how the resulting profiles relate to two further measures—teachers’ self-reported engagement in teaching CT and their intention to pursue further professional learning in CT pedagogy—and we complement the survey with interviews in which teachers describe the conditions under which they teach. This explanatory sequential design allows us to ask not only who is ready but how teachers account for what happens between commitment and classroom practice. The study addresses three research questions:

- RQ1. How many, and what kinds of, latent readiness profiles characterize primary EFL teachers with respect to teaching critical thinking?
- RQ2. How do these profiles differ in demographic composition and, above all, in their enacted classroom engagement in teaching CT relative to their intention to pursue further professional learning?
- RQ3. What conditions do teachers themselves identify as shaping whether their commitment to CT reaches the classroom?

## 2. Literature Review and Conceptual Framework

### 2.1. Critical Thinking and Its Cultivation

Although definitions of critical thinking vary, most converge on a dual structure: a set of cognitive skills—interpretation, analysis, evaluation, and inference—and a dispositional component, the consistent inclination to deploy those skills (Ennis, 1996; Facione, 2000). Halpern (1998) and Dwyer et al. (2014) argue that both components must be deliberately taught and that neither reliably emerges as a by-product of content coverage. Meta-analytic evidence supports this claim: explicit instruction combined with practice, dialog, and authentic problems yields robust gains in students’ reasoning (Abrami et al., 2015; Heijltjes et al., 2014). Crucially, however, these syntheses also show that effects hinge on how teachers design and mediate tasks, and that infusing CT into subject teaching is more sustainable than bolting it on as a separate module (Bezanilla et al., 2019). The pivotal variable, in other words, is teacher readiness.

### 2.2. Teaching Critical Thinking in Foreign-Language Classrooms

Foreign-language education is doubly implicated in the CT agenda. On one hand, language learning is intrinsically interpretive, offering natural openings to reason about meaning, perspective, and evidence (Alnofaie, 2013). On the other, primary EFL teachers face an acute version of the general tension: they must cultivate higher-order thinking while securing foundational language competence, frequently within examination regimes that reward recall over reasoning (Li, 2023; Zhang et al., 2020). Studies of EFL teachers’ conceptions consistently report broad endorsement of CT alongside uncertainty about enactment and doubts about young learners’ readiness (Li, 2023). A parallel pattern recurs in research on language-learner instruction, where teachers voice favorable attitudes toward accommodating English-language learners yet translate those attitudes unevenly into practice (Reeves, 2006). Such findings imply that favorable orientations are necessary but not sufficient, and that the field must move beyond documenting mean beliefs toward mapping the configurations of belief, capability, and constraint that differentiate teachers.

### 2.3. Teachers’ Beliefs, Efficacy, and Disposition

Decades of research show that teachers’ beliefs and sense of efficacy shape instructional choices, effort, and persistence (Klassen & Tze, 2014; Pajares, 1992). In language education, teacher cognition likewise mediates between curricular intentions and classroom practice (Borg, 2003; Yuan & Lee, 2014). Building on this tradition, we conceptualize readiness to teach CT as multidimensional, encompassing teachers’ attitudes toward CT, perceived capability to teach it, access to supportive resources and policies, and contextual constraints such as unsupportive norms and external barriers, which can impede critical thinking even among otherwise capable individuals (Dwyer, 2023). We additionally include teachers’ own CT disposition, a comparatively underexamined personal characteristic reflecting the habitual tendency to question assumptions, consider alternatives, and reason with evidence (Facione, 2000).

Including CT disposition extends readiness beyond what teachers believe about CT or perceive themselves able to do. Dispositions such as inquisitiveness, open-mindedness, and analyticity describe a person’s habitual orientation toward reasoning and are theorized to influence how information and problems are approached in everyday contexts (Facione, 2000; Halpern & Dunn, 2021). For teachers, such orientations may constitute a personal resource: those who habitually interrogate claims and weigh evidence may be more attentive to opportunities for eliciting similar reasoning from pupils. Yet being disposed to think critically is not equivalent to knowing how to teach critical thinking. A teacher may exhibit strong CT dispositions while lacking pedagogical knowledge, instructional confidence, resources, or supportive classroom conditions. Treating disposition as one component of readiness therefore makes it possible to examine whether personal orientation aligns with—or diverges from—teachers’ capacity and opportunity to enact CT instruction.

### 2.4. A Person-Centered Framework

Variable-centered analyses estimate average relations among variables across a population and therefore do not directly reveal whether similar outcomes arise from qualitatively different configurations of antecedents. Person-centered methods such as latent profile analysis (LPA) complement this approach by identifying subgroups characterized by distinct patterns across multiple attributes (Ferguson et al., 2020; Spurk et al., 2020). Applied to teachers, LPA has revealed meaningful profiles of wellbeing, striving, engagement, and burnout (Collie et al., 2015; De Clercq et al., 2022; Holmström et al., 2023; Virtanen et al., 2016), indicating consequential heterogeneity within teacher populations. This perspective has rarely been applied to readiness to teach critical thinking, particularly among primary EFL teachers.

**Figure 1:**
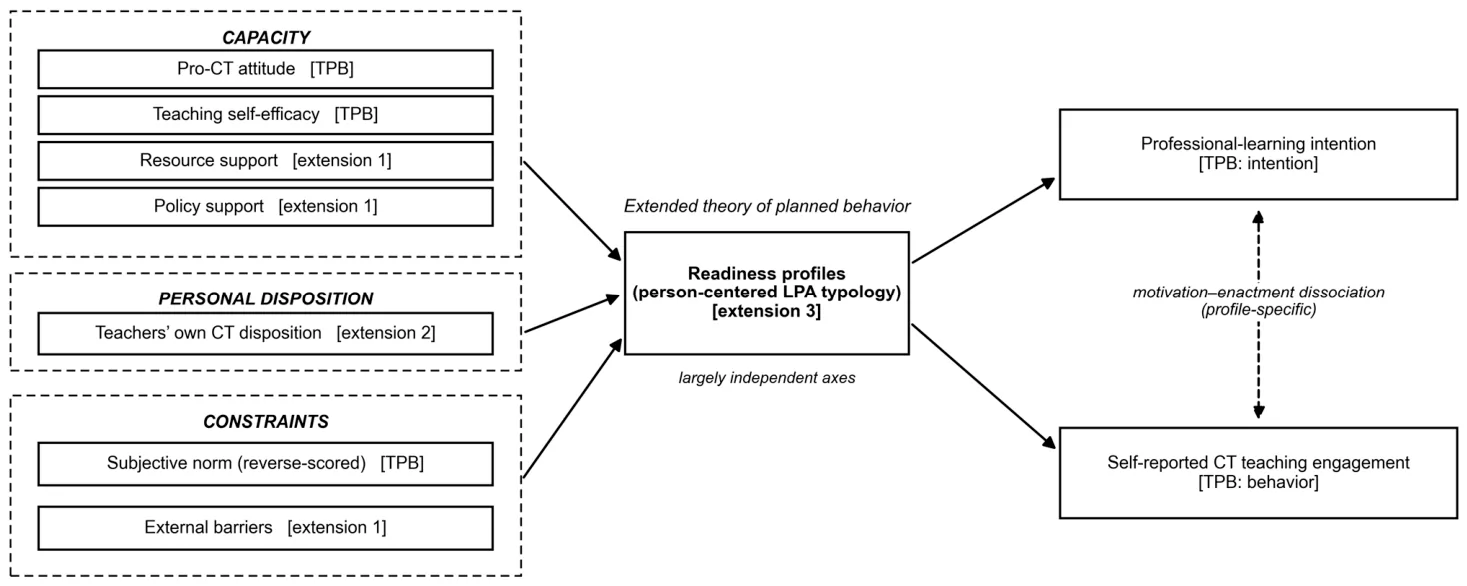
Conceptual framework: an extended, person-centered model of primary EFL teachers’ readiness to teach critical thinking. Components labeled [TPB] belong to the classic theory of planned behavior. Extension 1 models capacity separately from constraints rather than combining them as perceived behavioral control; extension 2 adds teachers’ own critical-thinking disposition as an antecedent; extension 3 represents readiness person-centered, as configurations rather than average effects. Subjective norm is measured with negatively worded items and reverse-scored, so higher values indicate a more supportive perceived norm. Capacity and constraint are hypothesized to form largely independent dimensions.

Figure 1 summarizes the conceptual framework guiding the study. The framework draws on the theory of planned behavior. In the classic model, attitude toward a behavior, subjective norm, and perceived behavioral control jointly shape intention, which in turn predicts behavior; perceived behavioral control may also act on behavior directly (Ajzen, 1991). The model has been applied to teachers’ instructional intentions in settings ranging from inclusive-education practice to technology integration (G. Liu & Wang, 2024; Sharma & Jacobs, 2016). Each element has a counterpart here. Attitude is teachers’ evaluation of teaching CT; subjective norm is their perception of whether CT teaching is expected and valued in their professional setting; and perceived behavioral control is teaching self-efficacy. Intention is the intention to pursue professional learning in CT pedagogy, and behavior is self-reported CT teaching engagement. The framework extends the model in three ways. First, the conditions that facilitate or impede enactment—resource support, policy support, and external barriers—are modeled separately rather than folded into a single measure of perceived control, because a teacher may feel capable while working in circumstances that make the behavior difficult. Second, teachers’ own CT disposition is added as an individual difference reflecting the habitual tendency to reason critically. Third, the model is represented as person-centered rather than variable-centered: the object of analysis is the configuration in which these elements co-occur within a teacher.

### 2.5. From Intention to Practice: The Enactment Gap

A recurring theme across the teacher-change literature is that favorable beliefs and intentions do not automatically translate into corresponding practice. Ajzen’s (1991) framework anticipates this possibility: intentions are more likely to be enacted when individuals possess sufficient control over the behavior and encounter conditions that permit its performance. Research on teachers documents similar discrepancies. Chen (2008), for example, found that teachers’ professed pedagogical beliefs about technology integration often diverged from their classroom practice because of a combination of external constraints, limited theoretical understanding, and competing beliefs. Comparable belief–practice gaps have also been reported where teachers endorse inclusive or learner-centered aims but enact them inconsistently in the classroom (Reeves, 2006). CT instruction may be particularly susceptible to such discrepancies because it requires teachers to create space for questioning, evidence evaluation, and extended reasoning while also meeting curricular and assessment demands. A person-centered design is therefore useful for examining whether intention and enactment align differently across teacher profiles and which combinations of personal and contextual conditions accompany any observed divergence.

## 3. Method

### 3.1. Design

An explanatory sequential mixed methods design was employed (quantitative → qualitative). The quantitative survey established the latent profiles and their correlates; a subsequent qualitative strand of semi-structured interviews illuminated the mechanisms underlying the profiles, with priority given to the quantitative phase and the qualitative strand used to explain and enrich it.

### 3.2. Participants

The survey was administered online in September and October 2024. Of 371 submitted responses, 30 that were completed in under 120 s—an indicator of careless responding—were removed, yielding 341 in-service primary EFL teachers. A second, content-based response-quality screen was then applied, described in Section 3.3, which removed a further 29 respondents who gave the identical answer to all 42 substantive items; the analytic sample is therefore 312. By IP address, respondents were located across three provinces and 12 cities but were overwhelmingly drawn from Shandong Province (99.4%), with the largest concentrations in Qingdao and Weifang. The sample was predominantly female (96.2%) and drawn mainly from urban schools (85.6%); teaching experience spanned early-career to veteran teachers, with the largest group (56.4%) reporting more than ten years of service, and most held a bachelor’s (86.2%) or master’s (13.5%) degree. Full demographic characteristics appear in Table 1. For the qualitative strand, five teachers (two urban, three rural; 1–6 years of experience; four holding a bachelor’s and one a master’s degree, with two pursuing further study) took part in semi-structured interviews conducted in December 2024, addressing their beliefs about CT, their classroom practices, and the constraints they encountered. Interviews were audio-recorded and machine-transcribed.

Recruitment used convenience sampling through regional primary-school teaching networks; participation was voluntary and anonymous, and teachers completed the questionnaire online. The five interviewees were purposively selected to vary in school location and career stage. Interviews followed a semi-structured protocol that moved from lesson-planning routines and resource use, through beliefs about the purpose of English teaching, to targeted probes concerning the elicitation of student reasoning, the handling of student questions, the obstacles teachers encountered, and the forms of support they desired. Each interview lasted approximately 30 to 45 min, was conducted remotely with consent, and was audio-recorded and machine-transcribed for analysis.

Ethical approval was granted by the Academic Ethics Committee of Qingdao University (approval no. QDU-PSY-20240907), and the study conformed to the Declaration of Helsinki. All survey respondents were informed of the study’s purpose and of the voluntary and anonymous nature of participation, were free to withdraw at any point, and provided informed consent before proceeding; no personally identifying information was collected, and IP addresses were used solely to summarize geographic distribution at the province and city level. The five interviewees gave separate consent for audio recording, and all transcripts were de-identified prior to analysis.

### 3.3. Response-Quality Screening

Speed-based screening alone provides a limited safeguard for a 42-item instrument, so response quality was also assessed at the content level using two established indices of response invariability (Curran, 2016): the longest string of consecutive identical responses, and the within-person standard deviation across the 42 substantive items. Twenty-nine respondents (8.5% of the initial 341) had a within-person standard deviation of exactly zero, having selected the same option for every item—27 choosing the midpoint throughout and two choosing 5 throughout. None of the 42 items is reverse-keyed, so this cannot be attributed to inconsistent item polarity; the set nevertheless spans nine constructs written in opposing directions, from favorable evaluations of CT teaching to statements that CT is not expected and that time and resources are lacking. Complete invariability across all of them was therefore treated as extreme straightlining, and these 29 cases were excluded. The analytic sample is 312, and its demographic composition is essentially unchanged from that of the unscreened sample (Table 1). A stricter alternative criterion—excluding the 46 respondents whose longest identical-response run covered at least half of the instrument—was retained as a sensitivity check rather than applied to the primary analysis; its results are reported in Section 4.4.

### 3.4. Measures

All multi-item constructs were measured on five-point Likert scales (1 = strongly disagree, 5 = strongly agree). Items were scored so that higher values represented more of the construct named by each scale; reverse-keyed items, where applicable, were recoded before scale scores were computed. Where suitable validated measures existed, items were adapted from established instruments; otherwise, items were developed for the present study. Before administration, the full item pool was reviewed by two education-research experts for content relevance and clarity, and wording was revised accordingly.

Seven indicators were used to characterize readiness to teach CT. Pro-CT attitude seven items, α = 0.98, ω = 0.98) captured teachers’ evaluations of incorporating CT into EFL instruction and its anticipated benefits for pupils, corresponding to the attitudinal component of the theory of planned behavior (Ajzen, 1991). Teaching self-efficacy (three items, α = 0.92, ω = 0.92) indexed confidence in implementing CT-oriented activities and was adapted from the teacher self-efficacy tradition (Tschannen-Moran & Hoy, 2001). Subjective norm (three items, α = 0.93, ω = 0.93) captured teachers’ perception of how far CT teaching is expected and valued in their professional setting. Its items are negatively worded (e.g., “Society does not expect primary EFL teachers to master CT pedagogy”) and were reverse-scored, so that higher values indicate a more supportive perceived norm. Perceived external barriers (three items, α = 0.91, ω = 0.91) captured pupil emphasis on results over process, curricular time pressure, and limited curricular space for independent thinking. Contextual support was administered as a single seven-item pool. Because factor analysis indicated two distinguishable dimensions (Section 3.4), it was scored as resource support (three items, α = 0.94, ω = 0.94), covering access to teaching materials, professional-development opportunities, school-level support, and policy support (four items, α = 0.86, ω = 0.86), covering both the perceived supportiveness of education policy for CT-oriented teaching and the extent to which teachers adjust their teaching to meet policy requirements. Teachers’ own CT disposition was assessed with items adapted from the California Critical Thinking Disposition Inventory (CCTDI; Facione, 2000; Facione et al., 1994).

The two distal outcomes correspond to the intention and behavior nodes of the framework. Self-reported CT teaching engagement (four items, α = 0.93, ω = 0.93) is the behavioral measure, and intention to pursue professional learning in CT pedagogy (three items, α = 0.91, ω = 0.91) is the intention measure; both were developed for the present study. Because they are self-reported rather than observed, they index teachers’ accounts of their practice rather than practice itself. Demographic covariates included gender, age, teaching experience, educational attainment, grade taught, school location, and familiarity with CT. Complete item wording, factor loadings, and reliability estimates for all measures developed for this study are reported in Supplementary Tables S1–S5.

The engagement items merit a brief note because they span both classroom practice and the teacher’s own intellectual activity: one asks directly about lesson design (“Even when it is not explicitly required, I take the initiative to design lesson content that develops critical thinking”), two about sustained engagement with demanding problems and habitual self-examination, and one about interest in CT pedagogy. The four cohere strongly (α = 0.93; inter-item *r* = 0.65 to 0.87, *M* = 0.76), and each item correlates more highly with the other three than with either CT disposition (*r* = 0.58 to 0.61) or professional-learning intention (*r* = 0.55 to 0.60), so the composite is not a restatement of those constructs. A measurement model that instead reassigned the two reasoning items to disposition and the interest item to intention fitted appreciably worse (CFI = 0.863 vs. 0.909; RMSEA = 0.101 vs. 0.082; SRMR = 0.070 vs. 0.054), and HTMT ratios were 0.71 with disposition and 0.70 with intention. We therefore retain the four items as a single measure of CT teaching engagement, and where a claim concerns classroom enactment specifically we report the single lesson-design item alongside it.

The disposition measure requires additional explanation because the administered CCTDI item set was reduced after psychometric evaluation. Nineteen items representing seven CCTDI facets were administered. Internal consistency varied substantially across facets. Analyticity (four items, α = 0.76), systematicity (two items, α = 0.81), self-confidence (three items, α = 0.86), and inquisitiveness (three items, α = 0.84) showed acceptable reliability, whereas cognitive maturity (two items, α = 0.38), truth-seeking (two items, α = 0.47), and open-mindedness (two items, α = 0.42) did not. A seven-factor CFA of the 19 items showed mixed fit, χ^2^(114) = 287.43, *p* < .001, CFI = 0.915, TLI = 0.885, RMSEA = 0.081, and SRMR = 0.069, with several weak loadings concentrated in the poorly performing facets. Both cognitive-maturity items and both truth-seeking items were reverse-keyed, as was one inquisitiveness item (“If possible, I try to avoid reading”), which also performed poorly. Because reverse-keyed items are known to be susceptible to wording-related method effects, this provides one possible explanation for their weak performance, although translation or construct-specific effects cannot be ruled out. The two positively worded open-mindedness items also showed limited coherence (*r* = 0.26).

On this basis, 12 items representing analyticity, systematicity, self-confidence, and inquisitiveness were retained. Their four-factor CFA showed improved comparative and residual fit, although RMSEA remained elevated, χ^2^(48) = 132.60, *p* < .001, CFI = 0.941, TLI = 0.918, RMSEA = 0.095, and SRMR = 0.073. The retained items yielded high composite reliability (α = 0.91, ω = 0.93). Because three original CCTDI facets were omitted, however, this measure should not be interpreted as reproducing the full CCTDI construct. It is used here as a reduced, CCTDI-derived indicator emphasizing four CT dispositions; all administered and excluded items are reported in Supplementary Table S3.

Having described the measures, we now report the evidence on which their scoring rests. Three analyses are presented in turn: an exploratory factor analysis of the readiness items, a confirmatory measurement model, and tests of discriminant validity. The exploratory analysis used maximum likelihood extraction with oblimin rotation. The 35 items entering the readiness composites were suitable for factoring (KMO = 0.92; Bartlett’s χ^2^(595) = 10,671, *p* < .001), and parallel analysis retained six factors accounting for 69.3% of the variance. The solution matched the intended dimensions with three departures. First, the seven contextual-support items separated into the resource-support and policy-support factors used throughout. Second, self-efficacy and resource-support items loaded on a common factor, indicating overlap between perceived capability and access to enabling resources. Third, two of the four analyticity items loaded more strongly on attitude than on disposition. Attitude, subjective norm, external barriers, and the remaining disposition items loaded as intended. All primary loadings exceeded the conventional criterion of 0.32 for a salient loading, ranging from 0.38 to 0.96; the complete pattern matrix appears in Supplementary Table S1. The second and third departures are taken up below and, respectively, in Section 4.4.

The confirmatory model quantifies what the exploratory solution indicated, using robust maximum likelihood (MLR) with the four retained CT-disposition facet scores as indicators of the disposition construct. Treating contextual support as one factor, as the questionnaire design assumed, fitted poorly, χ^2^(499) = 1612.22, CFI = 0.863, TLI = 0.846, RMSEA = 0.100, and SRMR = 0.093. Modeling resource support and policy support separately fitted substantially better, χ^2^(491) = 1241.09, CFI = 0.909, TLI = 0.896, RMSEA = 0.082, and SRMR = 0.054. The two therefore cannot be combined, and all subsequent analyses treat them as distinct indicators. Freeing six residual covariances between item pairs with closely overlapping wording improved fit further, χ^2^(485) = 1072.27, CFI = 0.930, TLI = 0.919, RMSEA = 0.073, and SRMR = 0.051; the unmodified nine-construct model remains the model of record. All standardized loadings were significant and ranged from 0.67 to 0.96, composite reliability from 0.86 to 0.98, and average variance extracted from 0.60 to 0.85 (Fornell & Larcker, 1981; Supplementary Table S4).

Discriminant validity was assessed with the HTMT criteria (Henseler et al., 2015). No ratio exceeded 0.90; the highest were self-efficacy with resource support (0.85), policy support with professional-learning intention (0.83), and attitude with engagement (0.83). The first pair required direct testing, not because the two constructs are conceptually close—one is a judgment about oneself and the other about one’s working conditions—but because the exploratory analysis had placed their items on a single factor. Modeling them separately fitted better than merging them, Δχ^2^(8) = 129.13, *p* < .001, ΔCFI = 0.017, and ΔBIC = 154, and their latent correlation was significantly below unity, *r* = 0.86, 95% CI [0.80, 0.93], and *z* = 4.27. They are therefore retained separately, and Section 4.4 reports what the profile solution looks like if they are combined. CT disposition was clearly distinct from self-efficacy (HTMT = 0.63; latent *r* = 0.65; two-factor CFI = 0.974 vs. 0.756 for one factor), and subjective norm was well separated from attitude (HTMT = 0.10), confirming that teachers’ own evaluation of CT and their perception of how it is regarded around them are not the same judgment.

Two measurement limitations follow from the instrument itself. No established external measure of CT-teaching resources or barriers was administered, so convergence with previously validated instruments could not be evaluated. In addition, the pro-CT attitude scale is highly redundant and reaches a ceiling: its seven items have a mean inter-item correlation of 0.85, and 54.8% of respondents obtained the maximum score, so the scale discriminates little among strongly pro-CT teachers. We retained all seven items rather than trimming them, because the redundancy is a property of the construct as measured rather than of particular items, and instead report in Section 4.4 the profile solution re-estimated without attitude altogether.

### 3.5. Common Method Variance

Because all constructs were measured by teacher self-report within a single questionnaire, common method variance (CMV) remains a potential source of bias (Podsakoff et al., 2003, 2012, 2024). Procedurally, participation was anonymous and voluntary, respondents were told that there were no right or wrong answers, and measures were separated within the questionnaire: the disposition block preceded the teaching-related measures, whereas professional-learning intention appeared at the end. These design features reduce some sources of evaluation apprehension and proximal consistency, but they do not eliminate same-source bias.

Three statistical diagnostics were therefore conducted, following recommended practice for single-source designs (Podsakoff et al., 2003, 2024). Harman’s single-factor test, a conventional rather than a definitive check, yielded a first unrotated factor accounting for 39.3% of item variance (41.6% for the first principal component). A one-factor confirmatory model fitted very poorly, χ^2^(819) = 5494.26, CFI = 0.491, RMSEA = 0.162, and SRMR = 0.140, indicating that a single common factor cannot reproduce the observed covariance structure. Most informatively, adding an unmeasured common latent factor orthogonal to the substantive factors (Podsakoff et al., 2012; Williams et al., 1989) left every substantive loading significant but reduced their mean standardized magnitude from 0.84 to 0.64, attributing approximately 30% of item variance on average to the method factor. Method-related variance is therefore nontrivial rather than absent.

One feature of the correlation matrix bears directly on how much of this matters. Absolute correlations among the readiness composites range from 0.03 to 0.79 (Supplementary Table S7), yet subjective norm and external barriers are almost unrelated to reported engagement (*r* = 0.07 and −0.01) and to the single enactment item (*r* = 0.08 and 0.01), even though all were collected from the same respondents in the same format. This is not a scoring artifact: item polarity was verified against the pre- and post-reverse-scoring data files, and reversing a scale changes the sign of a correlation, not its magnitude. Because a uniformly favorable response tendency would be expected to inflate associations among all self-reported measures alike, it is an unlikely sole explanation for a pattern in which some associations are strong and others near zero. This does not rule out common method variance, which need not affect all constructs equally (Podsakoff et al., 2024). We therefore interpret associations among self-reported measures cautiously, treat reported enactment as potentially inflated in absolute terms, and identify observational or multi-informant measurement as a priority for future work.

### 3.6. Analytic Strategy

Latent profile analysis was conducted in R using mclust on the seven standardized readiness indicators. One- through six-profile solutions were fitted under an equal-volume, diagonal covariance specification (mclust model EEI). Model selection considered BIC, sample-size-adjusted BIC, entropy, the bootstrapped likelihood-ratio test (BLRT; 1000 parametric bootstrap draws per adjacent-profile comparison), minimum profile size, and substantive interpretability (Nylund et al., 2007; Nylund-Gibson & Choi, 2018; Spurk et al., 2020). The analysis yields, for every teacher, a posterior probability of belonging to each profile rather than a definite assignment; how those probabilities were carried into the subsequent comparisons is described next. Interview transcripts were analyzed using a hybrid inductive–deductive thematic approach. Initial codes were generated from the transcripts by the first author and reviewed by the corresponding author, retaining participants’ own wording where analytically informative; related codes were then grouped into broader categories and themes. The survey constructs served as sensitizing concepts rather than a fixed coding scheme, so that themes not anticipated by the quantitative model could emerge.

The simplest way to use those probabilities is to assign each teacher to the most likely profile and then treat the assignment as observed, but doing so discards the uncertainty and can bias associations with external variables (Asparouhov & Muthén, 2014; Bolck et al., 2004; Vermunt, 2010). Associations between profile membership and the distal outcomes were therefore estimated in three ways. Primary estimates used the BCH procedure, which corrects class-specific estimates with weights derived from the estimated classification-error matrix and performs well for continuous distal outcomes (Bakk & Vermunt, 2016); standard errors came from a sandwich estimator accounting for teachers contributing weighted information to more than one profile. As a sensitivity analysis, the same contrasts were estimated from 50 pseudo-class draws combined by Rubin’s rules (Asparouhov & Muthén, 2014). Fifty draws were used here and in the covariate analysis below so that the two are directly comparable; the estimates were unchanged when 20 draws were used. Finally, conventional modal-assignment analyses—analysis of variance with Games–Howell comparisons, which assume neither equal variances nor equal group sizes (Games & Howell, 1976)—are reported to facilitate comparison with the wider person-centered literature, not as estimates of equal evidential standing.

Associations between demographic characteristics and profile membership were examined with the same error-adjusted logic: 50 pseudo-class draws were taken from the posterior probabilities, a multinomial logistic regression was fitted within each draw, and estimates were combined by Rubin’s rules, with Wald tests computed from the pooled coefficient vector and its total covariance matrix. The corresponding modal-assignment analysis is reported alongside for comparison. Coding of the covariates followed the substantive ordering of the response options: school location was ordered from urban to rural (urban, urban–rural fringe, township, rural), whereas grade taught was treated as nominal because “multiple grade levels” occupies no position on a grade-level continuum. Age, teaching experience, education, and CT familiarity were modeled as ordinal trends.

Finally, because several measurement decisions could reasonably have been made differently, the entire profile analysis was repeated under a series of alternative indicator, outcome, and screening specifications; these are reported with the results they qualify. Where a claim concerns classroom enactment specifically, it rests on the single item that directly assesses CT-oriented lesson design, with the four-item engagement composite reported alongside it.

No universally applicable sample-size threshold exists for latent profile analysis, because adequacy depends jointly on sample size, class separation, the number and quality of indicators, and the number and relative size of the profiles (Nylund et al., 2007; Tein et al., 2013). Simulation work shows that class recovery depends especially on profile separation, with the performance of BIC, BLRT, and related criteria varying across conditions rather than improving at a single cutoff (Tein et al., 2013). The analytic sample of 312 falls within the range commonly regarded as workable for applied LPA. We therefore evaluated precision directly for the comparisons that follow. For a contrast between two profiles of the sizes obtained here, a sensitivity analysis with α = 0.05 and power = 0.80 indicated a minimum detectable standardized mean difference of *d* = 0.51; for the demographic associations, the minimum detectable effects were *w* = 0.19 for a three-degree-of-freedom test and *w* = 0.22 for a nine-degree-of-freedom test. Associations of approximately moderate magnitude were therefore detectable, whereas small ones may not have been.

### 3.7. The Qualitative Strand: Scope and Trustworthiness

The five interviews were conducted to illuminate processes and contextual experiences that might help explain the quantitative pattern, and the scope of that contribution should be stated at the outset. The interviewees were purposively selected to vary in school location and career stage, and they are not classified into profiles by measurement. What the qualitative strand offers is not verification of profile membership but accounts in which the configurations identified quantitatively become recognizable: we examined whether an interviewee’s account combined strong commitment to CT with substantial normative or structural constraint, and whether it combined such commitment with limited professional and material support. Which accounts corresponded to which configuration is reported with the qualitative findings in Section 4.5. This correspondence is interpretive, and it is deliberately coarse: it uses only these two broad features, so that the more specific themes reported below were developed from the interview material rather than built into the correspondence itself. Purposeful qualitative sampling in mixed methods research may legitimately prioritize information-rich cases relevant to particular quantitative findings over proportional representation of every subgroup (Guetterman et al., 2015; Ivankova et al., 2006; Onwuegbuzie & Collins, 2007; Palinkas et al., 2015). With five participants, however, some configurations will inevitably go unrepresented, and the qualitative strand cannot speak to how teachers in less constrained circumstances enact CT.

Within these limits, several procedures supported analytic trustworthiness. Coding moved from low-inference, data-near codes—including participants’ own wording where informative—to progressively broader categories and themes, with an audit trail linking each stage of interpretation to the underlying transcript material. The first author developed the initial coding scheme, which the corresponding author then reviewed against the full transcripts; disagreements were resolved through discussion and renewed examination of the relevant source passages.

## 4. Results

### 4.1. Profile Enumeration

Fit indices for the one- through six-profile solutions are reported in Table 2. The BIC did not reach a minimum within the range examined: it fell by 151 points from three to four profiles, by only 5 points from four to five, and then by a further 235 points from five to six. The BLRT was significant at every adjacent comparison, including five versus six. Neither criterion therefore identifies a stopping point, and with continuous indicators a monotonically decreasing BIC is expected when within-class distributions depart from normality, in which case additional classes capture distributional features rather than substantively distinct subgroups (Bauer & Curran, 2003; Nylund-Gibson & Choi, 2018). Enumeration therefore rested on profile size, parsimony, substantive interpretability, and classification precision (Nylund et al., 2007; Tein et al., 2013; Spurk et al., 2020), and we present it as a judgment rather than as a criterion-driven result. Both the five- and six-profile solutions contained a profile of only 12 teachers (3.8% of the sample), too small in the present sample to support stable substantive interpretation or downstream comparisons. In the five-profile solution this additional profile emerged by subdividing the Empowered profile, isolating 12 teachers characterized by high barriers and low policy support. The six-profile solution added a further class of 21 teachers whose scores were near-uniform across constructs written in opposing directions (all means between 2.5 and 3.2), a pattern more readily attributed to response style than to a distinct configuration of readiness. By contrast, the four-profile solution retained substantively distinct groups of adequate size and showed high classification precision: entropy was 0.93, the smallest profile comprised 48 teachers (15%), the mean maximum posterior probability was 0.96, and average posterior probabilities for assigned profiles ranged from 0.91 to 0.97. We retained four profiles on these grounds while acknowledging that the information criteria alone would favor a larger number.

### 4.2. Profile Characterization

**Figure 2:**
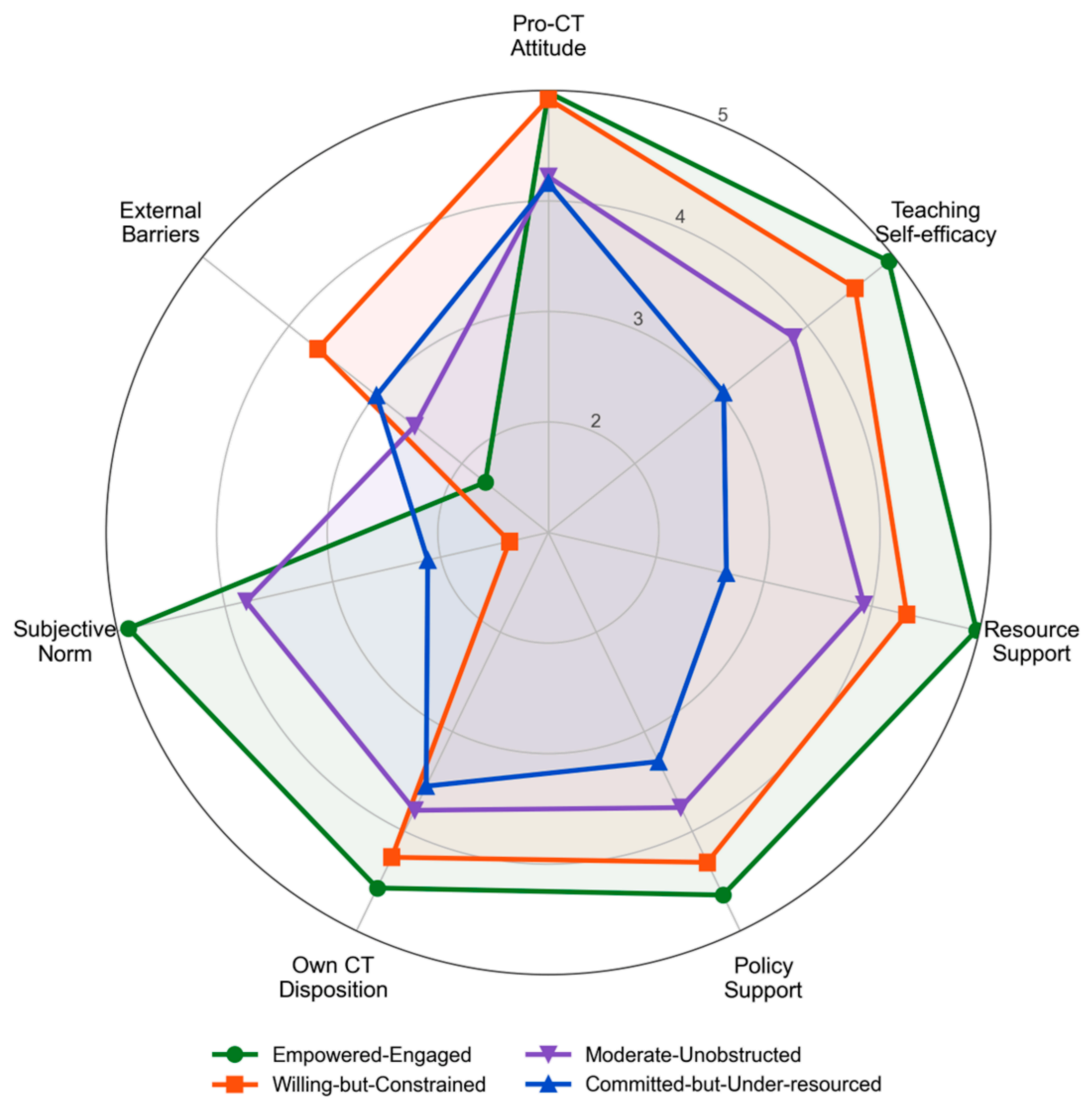
Radar comparison of the four profiles across the seven readiness dimensions (raw 1–5 scores). Subjective norm is reverse-scored, so higher values indicate a more supportive perceived norm. The Empowered-Engaged and Willing-but-Constrained profiles overlap on the capacity spokes but separate sharply on subjective norm and external barriers.

**Figure 3:**
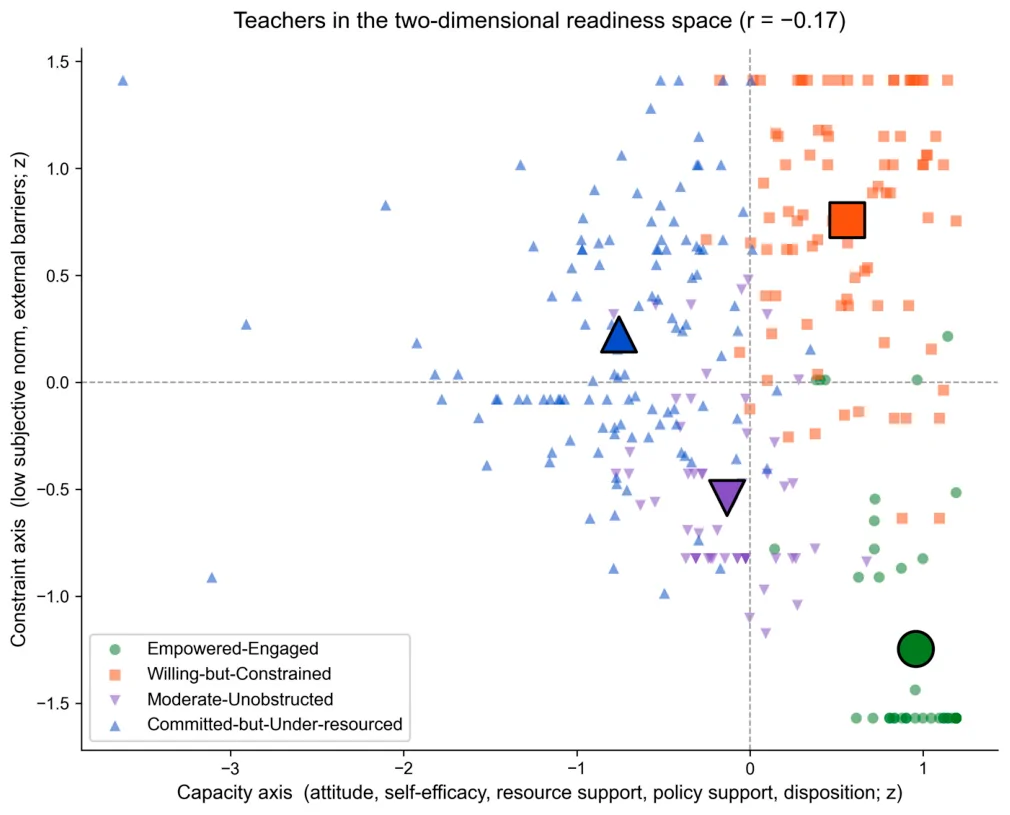
Individual teachers plotted on the capacity dimension (mean standardized attitude, self-efficacy, resource support, policy support, and disposition) and the constraint dimension (mean standardized reverse-scored subjective norm and external barriers), colored by profile. Large markers denote profile centroids; the two composites correlate *r* = −0.17.

The four profiles are described in Table 3 and displayed in Figure 2 and Figure 3. Empowered-Engaged teachers (n = 48, 15%) combined uniformly strong resources with an unobstructed setting: pro-CT attitude (4.98), teaching self-efficacy (4.94), resource support (4.97), policy support (4.64), and CT disposition (4.57) were all high, they perceived CT teaching as clearly expected and valued around them (subjective norm = 4.90), and external barriers were minimal (1.73). Willing-but-Constrained teachers (n = 88, 28%) were almost indistinguishable from them in commitment—pro-CT attitude differed by 0.06 points (4.92 vs. 4.98)—but occupied a very different context: they perceived little external expectation that CT should be taught (1.36) and reported the highest external barriers (3.67). Moderate-Unobstructed teachers (n = 61, 20%) also held favorable attitudes (4.22) with more moderate self-efficacy and resource support (3.84 and 3.93), in a relatively supportive and unobstructed setting (norm = 3.80; barriers = 2.55). Committed-but-Under-resourced teachers (n = 115, 37%), the largest profile, remained favorable toward CT (4.16) but combined the lowest self-efficacy (3.03) and resource support (2.65) with a weakly supportive perceived norm (2.12). No profile was defined by rejection of CT. What separated these teachers was not whether they valued critical thinking but what they had to work with and what their setting expected of them.

Figure 3 makes this structure explicit. For visualization, the five positively oriented indicators were standardized and averaged into a capacity dimension, and reverse-scored subjective norm and external barriers into a constraint dimension, both at the individual level. The two were only weakly related, *r* = −0.17, *p* = .003, sharing about 3% of their variance—so capacity and constraint are not opposite ends of one continuum. The profiles occupy four different combinations of them: high capacity with low constraint (Empowered-Engaged, *z* = 0.96 and −1.24), high capacity with high constraint (Willing-but-Constrained, 0.56 and 0.76), lower capacity with low constraint (Moderate-Unobstructed, −0.13 and −0.54), and lower capacity with higher constraint (Committed-but-Under-resourced, −0.76 and 0.22). A teacher may therefore possess considerable readiness and still work in circumstances that obstruct it.

In practical terms, the four profiles correspond to recognizable configurations of teacher readiness. The Empowered-Engaged represent the minority for whom strong commitment, perceived competence, supportive resources, and relatively few constraints coincide. The Committed-but-Under-resourced—the largest group—are similarly favorable toward CT but report substantially lower self-efficacy and resource support. Moderate-Unobstructed teachers occupy a different position: they encounter comparatively few constraints, yet their efficacy and other enabling conditions remain moderate rather than strong. The Willing-but-Constrained are the most distinctive configuration. They are nearly as favorable toward CT as the Empowered group and report high familiarity with CT, but simultaneously face the strongest normative and structural constraints. Their presence—more than one quarter of the sample—shows why readiness cannot be reduced to teacher willingness alone: strong commitment can coexist with conditions that make enactment difficult.

### 4.3. Predictors and Distal Outcomes

Demographic and background predictors of profile membership were examined with the classification-error-adjusted procedure described in Section 3.6, with the naive modal-assignment model reported alongside it (Table 4). Only one predictor was associated with profile membership under both approaches: the grade levels teachers taught, Wald = 71.81, *df* = 9, *p* < .001 after adjustment (LR χ^2^(9) = 21.83, and *p* = .009 under modal assignment). Teachers responsible for several grade levels at once were least common in the Empowered profile (18.8%, compared with 34.8–45.9% in the other profiles). This may reflect differences in teaching assignment or workload, but workload was not measured, so the interpretation cannot be resolved with the present data. Gender, age, teaching experience, education, and school location showed no detectable association under either approach (adjusted *p* = .25 to .94; descriptive Cramér’s V = 0.07 to 0.13). The two approaches diverged on one variable. Under modal assignment, CT familiarity appeared associated with membership, LR χ^2^(3) = 16.71, *p* < .001, and V = 0.19, with Willing-but-Constrained teachers reporting the highest familiarity (*M* = 3.78); after adjustment for classification uncertainty this association was no longer detectable, Wald = 3.35, *df* = 3, and *p* = .34. We therefore treat it as an artifact of assuming error-free assignment and do not interpret it further. The naive full model improved on the intercept-only model, LR χ^2^(27) = 48.40 and *p* = .007, with a pseudo-*R*^2^ of 0.058; because McFadden’s pseudo-*R*^2^ is not a proportion of explained variance, we interpret it only as indicating a modest overall improvement in fit. Sensitivity analysis indicated approximately 80% power to detect effects of *w* = 0.19 (3 df) and *w* = 0.22 (9 df); smaller associations may therefore have gone undetected, and these null results should not be taken to establish the absence of moderate associations.

On the distal outcomes, the profiles differed markedly on the enactment item, *F*(3, 308) = 59.0, *p* < .001, and η^2^ = 0.37, on the engagement composite, *F*(3, 308) = 75.3, *p* < .001, and η^2^ = 0.42, and on professional-learning intention, *F*(3, 308) = 43.9, *p* < .001, and η^2^ = 0.30. Games–Howell comparisons showed that every pair of profiles differed on the enactment item (all *p* ≤ .001) and on the composite, whereas on intention every pair differed except one. That exception is the study’s focal comparison. The Willing-but-Constrained profile did not differ detectably from the Empowered profile in intention (*M* = 4.52 vs. 4.67), *t*(83.4) = 1.29, *p* = .20, *g* = 0.24, and 95% CI [−0.11, 0.60], yet fell below it on the enactment item (*M* = 4.66 vs. 5.00), *t*(87.0) = 5.88, and *p* < .001, and on the engagement composite (*M* = 4.68 vs. 5.00), *t*(87.0) = 7.23, and *p* < .001. Two qualifications apply to these effect sizes. First, all 48 Empowered teachers gave the maximum response on the enactment item, so that group has zero within-group variance and the pooled-*SD* effect size (*g* = 0.77) is inflated by that compression; the distribution-free equivalents are more appropriate and more modest, Mann–Whitney *p* < .001, Cliff’s δ = 0.31, with 100% versus 69.3% of teachers at the scale maximum, χ^2^(1) = 16.50, and *p* < .001. Second, the absence of a detectable intention difference is not evidence of equivalence. A two one-sided tests procedure could not establish equivalence even against a liberal bound of *d* = 0.50 (*p* = .090), so the intention result is reported throughout as the absence of a detectable difference rather than as equality. With that wording, the pattern localizes the readiness gap: whatever is associated with these teachers’ lower reported practice is not accompanied by lower willingness to learn.

The Empowered and Willing-but-Constrained profiles should not, however, be treated as equivalent on every indicator. Because these indicators were used to derive the profiles, the contrasts below are descriptive; we report means and effect sizes rather than treating their significance as corroboration. The two profiles were closest in pro-CT attitude (4.98 vs. 4.92, Hedges’ *g* = 0.28) and policy support (4.64 vs. 4.31, *g* = 0.46), with larger differences in CT disposition (4.57 vs. 4.26, *g* = 0.56), teaching self-efficacy (4.94 vs. 4.55, *g* = 0.96), and especially resource support (4.97 vs. 4.33, *g* = 1.26). The two constraint indicators separated them further: by *g* = 1.54 for external barriers, and almost completely for subjective norm, where the profile means (4.90 vs. 1.36) sit near opposite ends of the scale with within-group *SD*s of 0.38 and 0.50. We describe the latter as a near-complete separation rather than report a standardized effect size, which that compression of within-group variance would inflate. Perceived constraint is thus the most distinctive difference between the two profiles, but not the only one: the substantial gap in resource support cautions against attributing the lower reported enactment of the Willing-but-Constrained group to constraint alone. Because the profiles are multivariate configurations rather than experimentally isolated conditions, the design cannot identify which component, or combination, accounts for that difference.

The comparisons above assign each teacher to a single profile. Repeating them with methods that carry the classification uncertainty forward changes none of the conclusions. Under the BCH procedure the profiles differed on the enactment item, Wald χ^2^(3) = 446.2, *p* < .001, the engagement composite, Wald χ^2^(3) = 554.8, *p* < .001, and professional-learning intention, Wald χ^2^(3) = 132.6, *p* < .001. In the focal contrast, Empowered teachers again reported higher enactment (difference = 0.34, *SE* = 0.06, *z* = 5.64, *p* < .001) and higher engagement (difference = 0.32, *SE* = 0.05, *z* = 6.92, *p* < .001), with no detectable difference in intention (difference = 0.15, *SE* = 0.13, *z* = 1.22, *p* = .22). Fifty pseudo-class draws pooled by Rubin’s rules gave the same pattern with wider standard errors: enactment difference = 0.33, *z* = 2.57, *p* = .010; engagement difference = 0.32, *z* = 2.87, *p* = .004; and intention difference = 0.15, *z* = 1.13, *p* = .26. In short, the contrast between what these teachers say they intend and what they say they do is not an artifact of treating profile membership as certain.

### 4.4. Robustness of the Typology

Several of the measurement decisions described in Section 3.4 could reasonably have been made differently. To establish whether the typology depends on them, the profile solution was re-estimated five times, each time altering one decision, and each solution was compared with the primary one by the percentage of teachers assigned to the corresponding profile (Supplementary Table S6). Four of the five changed almost nothing. Combining resource and policy support into a single indicator reproduced the solution closely (92.9% agreement, κ = 0.90); restricting disposition to the nine items that did not cross-load on attitude was essentially identical (99.0%, κ = 0.99); and omitting disposition (98.1%, κ = 0.97) or attitude (96.2%, κ = 0.95) made little difference. The attitude specification matters most, because attitude correlates 0.78 with the engagement composite: if the profile–outcome association were simply an artifact of building the profiles from a variable closely related to the outcome, removing attitude should dissolve it. It does not. The fifth change was consequential: merging self-efficacy with resource support produced a profile of only eight teachers (2.6%) and agreement of 61.2% (κ = 0.50), which does not prove the two constructs are distinct but does show that collapsing them yields a different and less stable typology. Across all five, the focal pattern held—there was no detectable intention difference between the two most capable profiles (*p* = .11 to .36) alongside a consistent enactment difference (all *p* < .001). One further specification behaves differently and is treated separately in Section 4.6.

The preceding checks varied how the profiles were built. Three further checks varied how the outcomes were defined and how the sample was screened, since both were also matters of judgment. First, the two reflection items were moved from the engagement composite to the disposition indicator, on the view that they describe the teacher’s own thinking rather than classroom activity; the solution barely moved (98.1% agreement, κ = 0.97) and the focal pattern held (intention *p* = .56; enactment *p* < .001). Second, the engagement scale’s interest item was added to the intention outcome. This is the one change that alters the intention result: the two focal profiles then differed, *t*(91.8) = 2.02 and *p* = .046. We report it because it marks a real boundary. The absence of a detectable intention difference is robust to how the profiles are specified but sensitive to where the interest item is placed, and the measurement model places it with engagement (Section 3.4). Third, the stricter screening criterion was applied, removing the 46 respondents with a run of at least 21 identical responses instead of the 29 with no within-person variance. This yielded four substantively similar profiles (n = 295; 17%, 28%, 20%, and 35%) and the same focal pattern (intention *t*(90.5) = 1.19, *p* = .24; enactment *t*(81.0) = 6.13, *p* < .001). Screening in the opposite direction is equally informative: retaining the 29 invariant responders produces an additional profile scoring at the midpoint on every dimension, composed disproportionately of those respondents, which is a signature of undifferentiated responding rather than a readiness configuration.

### 4.5. Qualitative Findings

Coding of the five interviews yielded six themes (Table 5). Applying the correspondence described in Section 3.7, three accounts contained the combination of strong commitment with substantial normative and structural constraint that characterizes the Willing-but-Constrained configuration (Teachers G, Y, and L), and two combined that commitment with limited professional and material support, as in the Committed-but-Under-resourced configuration (Teachers R and Z). These accounts therefore speak to the circumstances of the two configurations that together account for 65% of the survey sample. In the remaining two interviews we did not find passages that expressed the combinations characterizing the Empowered-Engaged or Moderate-Unobstructed configurations; this reflects what these five teachers described, not a judgment that no such teachers exist. Pseudonymous initials are used throughout.

T1: Shared endorsement of critical thinking.

Across the five interviews, endorsement of CT was unequivocal. All five teachers affirmed its educational value and rejected an exam-only conception of English teaching. Teacher G argued that “habits matter more than grades,” while Teacher L maintained that “critical thinking can be infused into every subject.” These accounts are consistent with the survey pattern, in which pro-CT attitudes were uniformly high across profiles, and with the broader literature showing that favorable pedagogical beliefs do not necessarily translate into corresponding classroom practice (Pajares, 1992; Reeves, 2006). Taken together, the qualitative and quantitative evidence suggests that endorsement itself is unlikely to be the principal dimension separating teachers’ readiness to teach CT.

T2: Limited pedagogical capacity for CT.

What teachers described was not a lack of commitment but uncertainty about how to translate CT into teachable classroom activity. Teacher Y referred to CT instruction as exceeding her current “reserve of knowledge,” while Teachers L and Z characterized their own competence as only “moderate” or “average.” None recalled substantial preparation in CT pedagogy during pre-service education. These accounts point to limitations in perceived capability and CT-specific pedagogical knowledge rather than unfavorable attitudes. They are consistent with research showing that teachers’ cognition and self-efficacy shape whether curricular intentions can be translated into classroom action (Borg, 2003; Tschannen-Moran & Hoy, 2001). They also echo the quantitative finding that strong endorsement of CT can coexist with lower self-efficacy and uneven access to professional support.

T3: Structural and normative constraints on enactment.

Teachers described a recurrent set of external and social constraints that narrowed the space available for CT-oriented instruction. Examination pressure was characterized by Teacher G as “an invisible cage” in which “everyone silently watches the scores.” Time and workload also recurred: Teacher L reported that English “class hours shrink year by year,” while Teacher Y, responsible for four classes, felt unable to reach pupils individually. Normative resistance to questioning was similarly salient. Teacher G estimated that “over 60% of teachers dislike students’ questions,” partly because they fear being unable to answer them. Importantly, these constraints were described alongside strong endorsement of CT rather than in place of it. The pattern therefore parallels the survey finding that favorable orientations and substantial obstacles can coexist within the same readiness configuration, and it is consistent with prior evidence that institutional and contextual barriers can disrupt the translation of pedagogical beliefs into practice (Chen, 2008).

T4: Commitment did not ensure sustained enactment.

Several teachers described initiating CT-oriented activities but curtailing, postponing, or abandoning them when classroom demands intervened. Teacher G recalled attempting a picture-prompt reasoning task whose “effect was very poor” and acknowledged that he would discontinue it. Teacher L deferred a pupil’s probing question until “after class” but, when asked whether she later returned to it, answered, “honestly, no.” Teacher Y avoided a dialectical discussion of a story villain because she feared “giving pupils the wrong impression.” These episodes illustrate how favorable orientations toward CT may coexist with incomplete or fragile enactment when teachers perceive limited control over classroom conditions. They provide a qualitative analog to the quantitative pattern in which the Willing-but-Constrained profile reported persistently high professional-learning intention alongside lower enactment and engagement, without implying that the same mechanisms can be assumed for the survey respondents themselves (Ajzen, 1991).

T5: Demand for CT-specific professional learning.

Rather than expressing resistance to CT, teachers repeatedly requested concrete and practice-oriented forms of support. Teacher Y stated that she “really needs to learn this,” while others called for access to “subject-matter experts,” model lessons, and training grounded in realistic classroom cases. Their requests were not for general encouragement to value CT, but for guidance on how to design questions, structure activities, manage time, and respond to pupils’ reasoning in practice. Such preferences correspond closely to established features of effective professional development, particularly content focus, active learning, and sustained opportunities to connect new ideas with classroom practice (Desimone, 2009). The interviews therefore help explain why willingness to develop CT pedagogy may remain high even among teachers who experience substantial constraints: the perceived need is not persuasion, but usable pedagogical support.

T6: Reflective orientations as a personal resource.

Finally, several teachers described practices suggestive of reflective and inquisitive orientations toward their own teaching. Teacher Y characterized her continual revision of materials as “endless self-negation, then trying new angles,” while Teacher L recorded unresolved post-class questions so that she could revisit them later. Such accounts resonate with conceptions of CT disposition as a habitual tendency to question assumptions, consider alternatives, and reflect on the adequacy of one’s reasoning (Ennis, 1996; Facione, 2000). In the present context, these orientations may function as a personal resource by making teachers more attentive to uncertainty, alternative interpretations, and opportunities for pupil reasoning. At the same time, the interviews also underscore that reflective disposition is not equivalent to pedagogical competence: teachers may habitually question and reconsider their own practice while still lacking the specialized knowledge, confidence, resources, or contextual latitude required to teach CT effectively.

### 4.6. How Far the Focal Contrast Depends on the Subjective-Norm Indicator

One specification deserves separate treatment because it does not reproduce the focal pattern. The Empowered-Engaged and Willing-but-Constrained profiles are distinguished above all by subjective norm: the first perceives CT teaching as expected and valued in its setting (*M* = 4.90); the second does not (*M* = 1.36), while the two are close on attitude, self-efficacy, and disposition. It is worth asking what remains of the contrast if that indicator is set aside.

Re-estimating the profiles from the remaining six indicators again produced two high-capacity groups, one reporting few external barriers (n = 48, *M* = 1.15) and one reporting many (n = 57, *M* = 4.16). Their outcomes reversed the primary pattern: professional-learning intention differed (4.81 vs. 4.49, *t*(101.2) = 2.52, *p* = .013, *g* = 0.48) while reported enactment did not (4.92 vs. 4.95, *p* = .54). Teachers who differ mainly in the practical difficulty of their circumstances thus show a difference in willingness but not in reported practice, which is the opposite of the profile contrast reported in Section 4.3.

Two further observations bear on how to interpret this. First, neither obstacle measure predicts the outcomes on its own (HTMT = 0.02 to 0.08), so the contrast in Section 4.3 arises from a configuration of indicators rather than from perceived constraint acting as a single variable. Second, because 42.6% of respondents combined strongly favorable attitudes with a weakly supportive perceived norm, we examined whether that combination reflects a general tendency to agree rather than a substantive position. It does not: an acquiescence index formed from the standardized attitude and norm scores was higher in the Willing-but-Constrained profile (0.73) than in the others (−0.60 to −0.05), yet controlling for it left the enactment difference intact and in fact larger (unadjusted *b* = 0.34, adjusted *b* = 1.06, both *p* < .001), with intention undetectable throughout (*p* = .18 and .12).

Taken together, these analyses mark the boundary of the focal claim. The typology and the contrast reported in Section 4.3 stand, and they survive the response-style explanation that would most obviously dissolve them. What they do not support is a general proposition that perceived constraint accompanies undiminished willingness and reduced practice. That pattern is specific to configurations defined to include how teachers perceive CT to be regarded around them, and replication with a balanced-keying norm instrument would be required before it could be treated as established.

## 5. Discussion

This study identified four profiles of primary EFL teachers’ readiness to teach critical thinking (CT). The profiles are organized by two weakly related dimensions: the personal and material capacity teachers bring to CT teaching, and the constraints they perceive in their setting. Interviews with five teachers gave qualitative substance to the two more constrained configurations. Three findings are consequential. Readiness is configural rather than a single continuum. Strong willingness can coexist with lower reported enactment, within limits set out below, and teachers’ own CT disposition contributes to readiness without defining it.

### 5.1. Readiness as Configurations of Capacity and Obstacles

The four-profile solution shows why readiness should not be understood as a continuum from “unready” to “ready.” Capacity and obstacles were only weakly associated (*r* = −0.17), allowing strong enabling conditions to coexist with either few or substantial perceived obstacles. Thus, Empowered-Engaged and Willing-but-Constrained teachers were both strongly oriented toward CT but differed sharply in the constraints they reported; Moderate-Unobstructed and Committed-but-Under-resourced teachers represented corresponding configurations at lower levels of capacity.

This pattern extends person-centered research on teacher heterogeneity (De Clercq et al., 2022; Holmström et al., 2023; Virtanen et al., 2016) to CT instruction. Conventional variable-centered analyses can identify average associations among attitudes, efficacy, support, and barriers, but they do not directly reveal how these conditions combine within teachers. The present profiles show that favorable conditions and obstacles are not simply opposite ends of one readiness dimension.

Equally important, no profile was characterized by rejection of CT. Attitudes were favorable across all four groups, suggesting that lack of endorsement is unlikely to be the principal source of variation in this sample. The more consequential differences concern whether teachers possess the capability, support, and contextual latitude to translate that endorsement into practice.

### 5.2. High Willingness Can Coexist with Constrained Enactment

The clearest contrast was between the Empowered-Engaged and Willing-but-Constrained profiles. Under the primary specification, both reported similarly high professional-learning intention, with no statistically detectable difference, yet the Willing-but-Constrained group reported substantially lower classroom enactment and CT-oriented engagement. This pattern persisted after accounting for classification uncertainty and across alternative specifications of the profile indicators.

The finding should not be interpreted as proving equal motivation or as establishing that obstacles cause lower enactment. The two profiles also differed in resource support, self-efficacy, and disposition, and the intention contrast showed some sensitivity to how the outcome was operationalized. The more defensible conclusion is that high willingness does not guarantee correspondingly high enactment. Teachers can remain strongly committed to developing CT pedagogy while working under conditions associated with less reported implementation.

The interviews help explain how such a pattern may arise. Teachers whose accounts resembled the constrained configurations described limited CT-specific preparation, examination pressure, restricted instructional time, heavy workloads, and norms discouraging questioning. Several recounted initiating CT-oriented activities but abandoning or postponing them when immediate classroom demands intervened. At the same time, they continued to affirm the value of CT and actively requested further professional development. These accounts echo longstanding evidence that favorable teacher beliefs do not automatically translate into practice when pedagogical and contextual conditions intervene (Chen, 2008; Pajares, 1992; Reeves, 2006).

The contribution, therefore, is not to locate a single causal barrier but to identify where the readiness-to-practice divergence is most visible: in a configuration where strong commitment coexists with substantial constraint and uneven enabling resources.

### 5.3. Teachers’ CT Disposition as a Readiness Resource

Teachers’ own CT disposition adds a less commonly examined element to readiness. Disposition was empirically distinguishable from teaching self-efficacy, indicating that teachers’ habitual orientation toward questioning, reflection, and evidence is not reducible to confidence in performing CT-related instructional tasks. Interview accounts of repeatedly revising materials, reconsidering assumptions, and recording unresolved questions provide qualitative examples of such orientations.

Its role should nevertheless be interpreted modestly. Removing disposition from the profile model left the solution almost unchanged, and the measure retained only four of the seven original CCTDI facets. Disposition therefore appears better understood as a supporting personal resource within readiness than as a necessary foundation for CT teaching. The finding extends teacher-cognition research by suggesting that teachers’ own intellectual orientations may matter for readiness, while leaving the magnitude and causal role of that contribution for future study.

### 5.4. Implications for Teacher Development

The findings shift the practical emphasis from persuading teachers that CT matters toward helping them act on commitments they already hold. Where pro-CT attitudes are widespread, generic advocacy is unlikely to address the principal difficulties teachers report. More useful interventions may combine CT-specific pedagogical development with changes to the conditions under which teachers work.

Professional development should therefore move beyond general discussion of CT and provide concrete support for designing reasoning tasks, questioning students, responding to unexpected answers, and balancing CT activities with curricular demands. Such content-focused and practice-based approaches are consistent with established principles of effective professional development, and a randomized field trial has shown that structured professional development produces measurable gains in teacher efficacy (Desimone, 2009; Ross & Bruce, 2007). The profiles also imply that support should be differentiated. Committed-but-Under-resourced teachers may benefit particularly from pedagogical resources and confidence-building opportunities, whereas Willing-but-Constrained teachers may require changes in instructional time, workload, assessment expectations, and school norms in addition to training.

At the policy level, the tension between CT-oriented curricular aspirations and examination-oriented accountability deserves particular attention. Where assessment continues to privilege coverage, accuracy, and recall, teachers have little room to sustain the questioning and exploratory reasoning that CT requires, and the teachers interviewed here described exactly this squeeze. Aligning what is assessed with what curricula claim to value is therefore a more promising lever than exhortation, and it is one that sits with system authorities rather than with individual teachers.

### 5.5. Limitations and Future Research

Several limitations bound these conclusions. First, the focal contrast depends on how perceived constraint was measured: it does not survive removal of the subjective-norm indicator, and that scale is negatively worded throughout, so a balanced-keying instrument is needed before the result can be regarded as established. Second, all quantitative variables are self-reported, and method-related variance was nontrivial—about 30% of item variance by the common-latent-factor estimate—so reported enactment may be inflated in absolute terms; observation, pupil reports, and instructional artifacts are needed to establish how the profiles relate to observed teaching. Third, the profiles are multivariate configurations whose focal groups differ in resources and self-efficacy as well as in perceived constraint, so the design cannot isolate which component accounts for the lower reported enactment; nor can four profiles be treated as the definitive number, since the information criteria continued to favor more classes and the solution was retained on grounds of size, interpretability, and classification precision. Fourth, the sample was geographically concentrated, predominantly female, and largely urban, and the five interviews spoke only to the two more constrained configurations; profile proportions should not be generalized beyond settings of this kind, and future explanatory sequential studies should recruit interviewees from within the measured sample so that all configurations can be given a voice. Finally, we were only able to locate five survey participants who agreed to contribute to our interview; future studies should attempt to invite more primary school teachers with varying demographic backgrounds and dispositional qualities to share their beliefs and practices.

## 6. Conclusions

Few aspirations in contemporary education are as widely endorsed, yet as unevenly realized, as the goal of teaching students to think critically. Whether that aspiration reaches the classroom depends substantially on teachers, but research has typically characterized them in the aggregate, estimating average associations of attitudes, efficacy, support, and constraints while revealing less about how these conditions coexist within different teachers. Profiling 312 primary EFL teachers across seven dimensions of readiness, and drawing on interviews with a separate sample of five teachers to illuminate selected configurations, this study shifted the question from how ready the average teacher is to what forms of readiness coexist within the profession. It also extended the concept of readiness beyond beliefs about CT teaching by considering teachers’ own disposition toward critical reasoning as one potential personal resource.

Four profiles emerged, organized by two weakly related dimensions: the capacity teachers bring to CT teaching and the constraints they perceive around them. Strong capacity could coexist with either few or many perceived constraints, and more modest capacity with either. No profile rejected CT, and among the characteristics examined only the grade levels teachers taught distinguished the profiles. Readiness is therefore better understood as a configuration of commitment, perceived capability, support, and constraint than as a single continuum from unwilling to willing. Substantively, this yields a typology where much of the literature has offered average effects; methodologically, it shows what a person-centered analysis reveals that a variable-centered one leaves implicit.

The typology also changes the practical question. Teachers in the Willing-but-Constrained profile wanted further professional learning as much as their Empowered peers did, yet reported teaching CT less often—a divergence that persists under methods accounting for classification uncertainty, though it holds within the measurement conditions set out above rather than universally. Where favorable attitudes toward CT are already near-universal, further advocacy is therefore unlikely to reach the difficulty teachers actually describe. What may reach it are the conditions under which they work: CT-specific preparation, usable materials, time for reasoning-oriented activity, assessment demands, and the norms surrounding student questioning. These are targets worth testing, not causes established here. The question for the next study is not whether primary EFL teachers value critical thinking—they do—but which forms of support allow that commitment to become ordinary classroom practice.

## Figures and Tables

**Table 1:** Sample demographic characteristics (N = 312).

Variable	Category	n	%
Gender	Female	300	96.2
	Male	12	3.8
Age	21–25	16	5.1
	26–30	47	15.1
	31–40	120	38.5
	41–50	83	26.6
	51–60	46	14.7
Teaching experience	1–3 years	52	16.7
	4–6 years	50	16.0
	7–10 years	34	10.9
	>10 years	176	56.4
Education	Associate	1	0.3
	Bachelor	269	86.2
	Master	42	13.5
Grade taught	Grades 1–2	55	17.6
	Grades 3–4	83	26.6
	Grades 5–6	66	21.2
	Multiple grades	108	34.6
School location	Urban	267	85.6
	Urban–rural fringe	19	6.1
	Township	17	5.4
	Rural	9	2.9
CT familiarity	Not at all familiar	1	0.3
	Unfamiliar	48	15.4
	Neutral	76	24.4
	Familiar	168	53.8
	Very familiar	19	6.1

Note. N = 312. All respondents taught English at the primary level. Percentages may not sum to 100 due to rounding. Grade taught is entered as a nominal factor in those analyses because one category is “several grade levels at once”.

**Table 2:** Fit indices for one- to six-profile solutions.

Profiles	LogLik	BIC	SABIC	Entropy	BLRT *p*	Smallest %
1	−3096	6271	6227	1.00	—	100
2	−2841	5808	5738	0.81	<0.001	49
3	−2723	5618	5523	0.90	<0.001	28
4	−2624	5467	5346	0.93	<0.001	15
5	−2599	5461	5316	0.93	0.009	4
6	−2458	5226	5055	0.94	<0.001	4

Note: N = 312; seven standardized readiness indicators; class-invariant, diagonal-covariance models. The four-profile solution was retained. BLRT = bootstrapped likelihood-ratio test, estimated from 1000 parametric bootstrap draws per adjacent-class comparison; the reported *p* is the proportion of draws whose likelihood-ratio statistic equaled or exceeded the observed value.

**Table 3:** Profile means (SD) on readiness indicators and distal outcomes.

Indicator	P1 Empowered-Engaged (n = 48)	P2 Willing-but-Constrained (n = 88)	P3 Moderate-Unobstructed (n = 61)	P4 Committed-but-Under-Resourced (n = 115)	F	η^2^
Pro-CT attitude	4.98 (0.12)	4.92 (0.25)	4.22 (0.45)	4.16 (0.97)	36.2	0.26
Teaching self-efficacy	4.94 (0.25)	4.55 (0.46)	3.84 (0.40)	3.03 (0.58)	259.4	0.72
Resource support	4.97 (0.15)	4.33 (0.63)	3.93 (0.46)	2.65 (0.58)	287.8	0.74
Policy support	4.64 (0.75)	4.31 (0.69)	3.76 (0.58)	3.30 (0.67)	61.3	0.37
Own CT disposition	4.57 (0.61)	4.26 (0.51)	3.79 (0.48)	3.55 (0.66)	46.9	0.31
Subjective norm	4.90 (0.38)	1.36 (0.50)	3.80 (0.56)	2.12 (0.87)	386.7	0.79
External barriers	1.73 (1.31)	3.67 (1.22)	2.55 (0.90)	2.99 (1.01)	34.7	0.25
Learning intention (distal)	4.67 (0.72)	4.52 (0.61)	3.84 (0.54)	3.54 (0.89)	43.9	0.30
CT engagement composite (distal)	5.00 (0.00)	4.68 (0.41)	4.08 (0.46)	3.65 (0.88)	75.3	0.42
CT lesson design, enactment item (distal)	5.00 (0.00)	4.66 (0.54)	4.10 (0.51)	3.60 (1.02)	59.0	0.37
CT familiarity (covariate)	3.31 (0.97)	3.78 (0.81)	3.41 (0.80)	3.41 (0.77)	5.1	0.05

Note: N = 312; *df* = 3308. All *F* tests are significant at *p* < .001 except CT familiarity (*p* = .002). Welch tests agreed with every *F* test for which they are defined; they are undefined for the two engagement measures because the Empowered profile has zero variance on both. Subjective norm is reverse-scored, so higher values indicate a more supportive perceived norm. The last four rows are distal outcomes and a covariate rather than profile indicators; the enactment item is the single engagement item referring directly to classroom practice.

**Table 4:** Demographic predictors of profile membership (multinomial logistic regression, *N* = 312).

Predictor	LR χ^2^	df	*p*
Gender	1.74	3	.628
Age	6.07	3	.108
Teaching experience	5.66	3	.129
Education	0.43	3	.934
Grade level taught	21.83	9	.009
School location	1.90	3	.593
Familiarity with CT	16.71	3	<.001

Note: Adjusted tests are Wald tests on estimates pooled across 50 pseudo-class draws by Rubin’s rules; likelihood-ratio tests compare the full modal-assignment model with the model omitting each predictor. Cramér’s *V* is computed from the bivariate cross-tabulation with profile membership. Coding of grade taught and school location follows Section 3.6.

**Table 5:** Exemplar coding table: from verbatim data to themes (five interviews).

Representative Quote (Translated from Chinese)	Open Code	Axial Category	Theme
“I also think it is important”; “I don’t quite agree” (with an exam-only focus)	Affirms CT value; rejects exam-only focus	Pro-CT endorsement	T1 Near-universal endorsement
“I don’t have that much of a reserve”; “moderate”; “hands-on practice is hard”	Low efficacy; insufficient knowledge base	Capability–knowledge gap	T2 Capability gap
“an invisible cage; everyone just silently watches the scores”	Exam culture suppresses CT	Exam-oriented norm	T3 Structural/normative obstacles
“class hours shrink year by year”; “I teach four classes”	Insufficient time; oversized classes	Structural constraints	T3 Structural/normative obstacles
“over 60% of teachers dislike children asking questions”	Collegial/disciplinary norms suppress questioning	Suppressive collegial norms	T3 Structural/normative obstacles
“the effect was very poor… I would give up”; “followed up after class… honestly, no”	Attempt then abandon; no follow-up	Attempt-then-abandon	T4 Motivation–enactment gap
“I really need to learn this”; “best to have subject-matter experts… training”	Demand for CT-specific PD	Demand for CT-specific PD	T5 Strong learning intention
“endless self-negation, then trying new angles”; recording post-class confusions	Reflective/self-critical disposition	Reflective disposition	T6 Own CT disposition

Note: Interview quotes were translated from the original Chinese transcripts; N = 5 interviews. The five interviewees were recruited separately from the survey and are not classified into profiles by measurement (Section 3.7).

## Data Availability

Data will be made available upon reasonable request addressed to first author.

## Supplementary Materials

The following supporting information can be downloaded at: https://www.mdpi.com/article/10.3390/jintelligence14090215/s1, Table S1: Complete Exploratory Factor Analysis Pattern Matrix (35 Readiness Items, N = 312); Table S2: Item Wording (Translated from Chinese); Table S3: CCTDI Items Excluded from the Disposition Indicator; Table S4: Reliability and Convergent Validity; Table S5: Heterotrait–Monotrait Ratios of Correlations; Table S6: Robustness of the Four-Profile Solution and of the Focal Contrast; Table S7: Correlations Among Composite Scores (N = 312).
