# Supplementary material for: From Willingness to Practice: A Person-Centered Study of Primary Teachers’ Critical-Thinking Readiness and the Enactment Gap
Source: J Intell. 2026 Sep 6;14(9):215. doi: 10.3390/jintelligence14090215 (PMC13608479; doi:10.3390/jintelligence14090215)
Supplement: Supplementary file 1 [file jintelligence-14-00215-s001.zip › jintelligence-4493279-supplementary.pdf]

Supplementary Materials

From Willingness to Practice: A Person-Centered Study of Primary Teachers' Critical-Thinking Readiness and the Enactment Gap

Table S1. Complete Exploratory Factor Analysis Pattern Matrix (35 Readiness Items, *N* = 312).

Scale	Item	F1	F2	F3	F4	F5	F6	Primary
Pro-CT attitude	AA1	.93	.05	.01	−.06	−.08	−.08	F1
Pro-CT attitude	AA2	.96	−.01	.07	−.09	−.04	−.06	F1
Pro-CT attitude	AA3	.96	.00	.03	−.08	−.04	−.06	F1
Pro-CT attitude	AA4	.82	.00	.15	.02	−.01	−.01	F1
Pro-CT attitude	AB1	.86	.01	.00	.09	.04	.07	F1
Pro-CT attitude	AB2	.91	−.04	−.02	.10	.09	.10	F1
Pro-CT attitude	AB3	.86	−.01	.00	.12	.05	.09	F1
Teaching self-efficacy	CA1	.13	.10	.66	.18	.03	.05	F3
Teaching self-efficacy	CA2	.14	.18	.50	.20	.03	.02	F3
Teaching self-efficacy	CA3	.10	.11	.57	.16	−.02	−.08	F3
Resource support	CD1	.01	.07	.84	−.03	.11	−.02	F3
Resource support	CD2	.03	.04	.92	.00	−.01	−.06	F3
Resource support	CD3	.04	−.02	.85	.09	.05	.09	F3
Policy support	CD5	.00	.03	−.04	.78	.00	−.14	F4
Policy support	CD6	−.02	−.04	.18	.79	−.02	.00	F4
Policy support	CD7	.05	.08	.21	.61	−.08	.01	F4
Policy support	CD8	.13	.07	.02	.50	.08	−.10	F4
Subjective norm (reverse-scored)	B2	−.01	.01	.07	.00	.81	−.07	F5
Subjective norm (reverse-scored)	B3	−.01	.00	.09	−.01	.91	.00	F5
Subjective norm (reverse-scored)	B4	.02	.03	−.07	−.02	.90	−.06	F5
External barriers	CB4	.03	.00	.04	−.02	−.17	.75	F6
External barriers	CB5	−.01	−.01	.01	−.07	.01	.89	F6
External barriers	CB6	−.01	.02	−.02	.00	−.02	.88	F6
Own CT disposition (analyticity)	Q14	.06	.38	−.10	.08	.17	.02	F2
Own CT disposition (analyticity)	Q15	.39	.30	−.17	.23	−.01	−.04	F1
Own CT disposition (analyticity)	Q16	.49	.32	−.23	.13	−.06	.00	F1
Own CT disposition (analyticity)	Q17	.35	.43	−.13	.14	−.02	.03	F2
Own CT disposition (systematicity)	Q18	.10	.61	−.01	.07	−.02	.00	F2
Own CT disposition (systematicity)	Q19	−.02	.67	−.01	.11	.00	.01	F2
Own CT disposition (self-confidence)	Q20	.06	.77	.08	−.10	.11	.00	F2
Own CT disposition (self-confidence)	Q21	−.07	.80	.08	−.02	.04	.01	F2
Own CT disposition (self-confidence)	Q22	−.04	.80	−.05	.06	.13	.03	F2
Own CT disposition (inquisitiveness)	Q23	.08	.67	.10	.00	−.15	−.10	F2
Own CT disposition (inquisitiveness)	Q24	−.10	.76	.21	−.03	−.07	−.02	F2
Own CT disposition (inquisitiveness)	Q25	.25	.59	−.02	.06	−.13	−.02	F2

Note. Maximum-likelihood extraction with oblimin rotation; parallel analysis retained six factors accounting for 69.3% of the variance. KMO = .92; Bartlett's $\chi^2(595) = 10{,}671$, $p < .001$. The three subjective-norm items are negatively worded and were reverse-scored before analysis, so that higher values indicate a more supportive perceived norm. F1 = pro-CT attitude (with analyticity items Q15 and Q16); F2 = own CT disposition; F3 = teaching self-efficacy together with resource support; F4 = policy support; F5 = subjective norm; F6 = external barriers. Primary loadings range from .38 to .96; the largest cross-loading is .35.

Table S2. Item Wording (Translated from Chinese).

Scale	Item	Item wording
Pro-CT attitude	AA1	Developing pupils' critical thinking improves their problem-solving ability.
Pro-CT attitude	AA2	Developing pupils' critical thinking increases their interest in learning.
Pro-CT attitude	AA3	Developing pupils' critical thinking increases their tolerance of cultural diversity.
Pro-CT attitude	AA4	Developing pupils' critical thinking improves their academic achievement.
Pro-CT attitude	AB1	Encouraging critical thinking can be applied even when teaching basic English skills.
Pro-CT attitude	AB2	Using activities that encourage pupils to think improves their language skills.
Pro-CT attitude	AB3	Integrating thinking skills into my English lessons helps pupils engage more with lesson content.
Teaching self-efficacy	CA1	I am confident about trying teaching activities that encourage pupils to think deeply.
Teaching self-efficacy	CA2	I can easily adapt my lessons to include activities that make pupils think.
Teaching self-efficacy	CA3	Even with methods I am unfamiliar with, I feel prepared to use approaches centred on pupils' thinking.
Resource support	CD1	I can obtain enough teaching materials to support activities that encourage pupils' critical thinking.
Resource support	CD2	I can access professional-development opportunities that help me learn how to engage pupils in thinking.
Resource support	CD3	My school gives me enough support (e.g., guidance, resources) to try new teaching methods that encourage pupils to think.
Policy support	CD5	I often adjust my teaching in order to comply with school and education-policy requirements.
Policy support	CD6	School and education policy give me adequate support to implement new teaching strategies.
Policy support	CD7	Education policy explicitly supports my including critical thinking and pupil discussion in class.
Policy support	CD8	Even when my personal view differs, I teach in line with education-policy requirements.
Subjective norm (reverse-scored)	B2	At the present stage of our country's development, developing pupils' critical thinking is not very important.
Subjective norm (reverse-scored)	B3	Critical thinking is not a core skill that an excellent English teacher must possess.
Subjective norm (reverse-scored)	B4	Society does not expect primary English teachers to master methods for teaching critical thinking.
External barriers	CB4	Pupils at my school care more about getting good results than about the process of learning.
External barriers	CB5	Time constraints in the curriculum make it hard for me to include activities focused on pupils' thinking.
External barriers	CB6	I feel the current curriculum leaves almost no space for activities in which pupils think independently.
Own CT disposition (analyticity)	Q14	It troubles me when people use superficial reasons to support a good position.
Own CT disposition (analyticity)	Q15	To object to someone else's view, one has to give appropriate reasons.
Own CT disposition (analyticity)	Q16	When handling a difficult problem, one should first work out where the crux of it lies.
Own CT disposition (analyticity)	Q17	Faced with a problem, I first identify what gave rise to it and then think about how to solve it.
Own CT disposition (systematicity)	Q18	I am good at drawing up systematic plans for solving complex problems.
Own CT disposition (systematicity)	Q19	I can sort out my own train of thought quite easily.
Own CT disposition (self-confidence)	Q20	Others usually appreciate my curiosity and desire to learn.
Own CT disposition (self-confidence)	Q21	I often come up with creative ideas.
Own CT disposition (self-confidence)	Q22	Because I can usually analyse problems calmly and objectively, my classmates and friends often ask my advice.
Own CT disposition (inquisitiveness)	Q23	Exploring new things makes my life richer.
Own CT disposition (inquisitiveness)	Q24	I look forward to taking on challenging tasks.
Own CT disposition (inquisitiveness)	Q25	When facing an important matter, I first do my best to gather all the relevant information.
CT teaching engagement	AC1	I habitually reflect on and examine my own actions and ideas.
CT teaching engagement	AC2	Even when it is not explicitly required, I take the initiative to design lesson content that

		develops critical thinking.
CT teaching engagement	AC3	I spend time exploring challenging problems in depth.
CT teaching engagement	AC4	I am interested in methods for teaching critical thinking and willing to explore and try new instructional strategies.
Professional-learning intention	CC1	I am interested in learning more about teaching methods that help pupils think independently.
Professional-learning intention	CC2	I am willing to attend training to learn how to encourage pupils' thinking in lessons.
Professional-learning intention	CC3	I would like to explore more professional-development opportunities focused on pupils' thinking skills.

Note. Item AC2 is the only engagement item that refers directly to classroom enactment; the analyses reported in Sections 4.3 and 4.4 of the manuscript use that item as the enactment indicator alongside the four-item composite.

Table S3. CCTDI Items Excluded from the Disposition Indicator.

Facet	Item	Item wording (translated from Chinese)
Cognitive maturity	Q9	When I face a controversial issue, I find it hard to choose one view among several. (reverse-keyed)
Cognitive maturity	Q10	If four reasons support a position and only one opposes it, I will go with the majority of reasons. (reverse-keyed)
Truth-seeking	Q11	Even when the evidence conflicts with my view, I stick to my own idea. (reverse-keyed)
Truth-seeking	Q26	I am unshakeably certain about the things I believe. (reverse-keyed)
Open-mindedness	Q12	It matters to me to know what different people think about the same thing.
Open-mindedness	Q13	Others have the right to express their opinions, but I will not necessarily adopt their view.
Inquisitiveness (reverse item)	Q27	If possible, I try to avoid reading. (reverse-keyed)

Note. Facet reliabilities: cognitive maturity α = .38, truth-seeking α = .47, open-mindedness α = .42. The retained facets were analyticity (α = .76), systematicity (α = .81), self-confidence (α = .86), and inquisitiveness (α = .84).

Table S4. Reliability and Convergent Validity.

Construct	k	α	ω	CR	AVE
Pro-CT attitude	7	.975	.981	.975	.848
Teaching self-efficacy	3	.916	.917	.913	.778
Resource support	3	.941	.942	.943	.846
Policy support	4	.857	.859	.858	.604
Subjective norm (reverse-scored)	3	.932	.932	.932	.821
External barriers	3	.906	.908	.908	.768
Own CT disposition (4 parcels)	12	.913	.934	.867	.623
CT teaching engagement	4	.926	.928	.930	.769
Professional-learning intention	3	.909	.912	.911	.774

Note. N = 312. CR = composite reliability; AVE = average variance extracted, both computed from the standardized solution of the respecified nine-construct confirmatory model (robust maximum likelihood): $\chi^2(491) = 1241.09$, CFI = .909, TLI = .896, RMSEA = .082, SRMR = .054.

Table S5. Heterotrait–Monotrait Ratios of Correlations.

Construct	ATT	EFF	RSUP	POL	SN	BARR	DISP	ENG	INT
ATT	1.00								
EFF	0.57	1.00							
RSUP	0.43	0.85	1.00						
POL	0.54	0.68	0.58	1.00					
SN	0.10	0.21	0.33	0.10	1.00				
BARR	0.04	0.08	0.08	0.22	0.54	1.00			
DISP	0.64	0.63	0.50	0.59	0.12	0.14	1.00		
ENG	0.83	0.77	0.66	0.67	0.03	0.02	0.71	1.00	
INT	0.61	0.65	0.49	0.83	0.02	0.07	0.64	0.70	1.00

Note. N = 312. ATT = pro-CT attitude; EFF = teaching self-efficacy; RSUP = resource support; POL = policy support; SN = subjective norm (reverse-scored); BARR = external barriers; DISP = own CT disposition; ENG = CT teaching engagement; INT = professional-learning intention. HTMT is computed from absolute correlations and is therefore unaffected by the reverse scoring. No ratio exceeds the lenient .90 criterion; three sit at or just below the conservative .85 criterion for conceptually distinct constructs (Henseler et al., 2015) and are discussed in Section 3.4 of the manuscript.

Table S6. Robustness of the Four-Profile Solution and of the Focal Contrast.

Specification	Profile sizes (%)	Entropy	Agreement (κ)	P1 vs. P2 intention	P1 vs. P2 enactment item
Seven indicators (reported solution)	15 / 28 / 20 / 37	.925	— (reference)	$p = .20$	$p < .001$
Resource and policy combined (six indicators)	14 / 32 / 20 / 34	.901	92.9% (.90)	$p = .12$	$p < .001$
Self-efficacy and resource support merged	18 / 40 / 40 / 3	.922	61.2% (.50)	$p = .11$	$p < .001$
Nine-item disposition indicator	15 / 28 / 20 / 37	.926	99.0% (.99)	$p = .25$	$p < .001$
Disposition indicator removed	16 / 28 / 20 / 37	.925	98.1% (.97)	$p = .36$	$p < .001$
Attitude indicator removed	14 / 28 / 20 / 39	.924	96.2% (.95)	$p = .18$	$p < .001$
Reflection items moved into the disposition indicator	16 / 29 / 18 / 37	.928	98.1% (.97)	$p = .56$	$p < .001$
Interest item added to the intention outcome	15 / 28 / 20 / 37	.925	identical solution	$p = .046$	$p < .001$
Stricter response-quality screen (n = 295)	17 / 28 / 20 / 35	.925	same four profiles	$p = .24$	$p < .001$
Subjective-norm indicator removed (six indicators)	26 / 41 / 15 / 18	.867	not comparable	$p = .013$	$p = .54$

Note. Agreement is the percentage of teachers assigned to the corresponding profile of the reported solution, with Cohen's κ in parentheses. Rows 2–6 and the final row vary the profile indicators; the remaining rows vary the outcome definition or the response-quality screen. Merging self-efficacy with resource support produces a degenerate solution with a class of eight teachers and is reported as a failed specification. The only row in which the focal intention contrast is not null is the one adding the interest item to the intention outcome; the measurement model assigning that item to engagement fits better (Section 3.4). In the final row, removing the subjective-norm indicator yields profiles that are not in one-to-one correspondence with the reported solution, so agreement is not comparable; the focal contrast is taken between the two highest-capacity profiles (n = 48 with barriers = 1.15 and n = 57 with barriers = 4.16). That specification reverses the focal pattern and is discussed in Section 4.6 of the manuscript.

Table S7. Correlations Among Composite Scores (N = 312).

Composite	ATT	EFF	RSUP	POL	SN	BARR	DISP	ENG	INT	AC2
ATT	1.00									
EFF	.54	1.00								
RSUP	.42	.79	1.00							
POL	.49	.60	.53	1.00						
SN	−.10	.20	.32	.10	1.00					
BARR	−.03	−.10	−.08	−.21	−.50	1.00				
DISP	.60	.58	.48	.52	.09	−.15	1.00			
ENG	.78	.71	.62	.60	.07	−.01	.65	1.00		
INT	.57	.59	.46	.74	−.01	−.08	.58	.64	1.00	
AC2	.74	.66	.57	.55	.08	.00	.58	.93	.60	1.00

Note. N = 312. AC2 = the single engagement item referring to classroom enactment; SN = subjective norm, reverse-scored so that higher values indicate a more supportive perceived norm. Absolute correlations among the seven readiness composites range from .03 to .79, whereas subjective norm and external barriers are essentially unrelated to CT teaching engagement and to the enactment item. This heterogeneity is difficult to reconcile with pervasive common method variance.
